# Locomotion of *C. elegans*: A Piecewise-Harmonic Curvature Representation of Nematode Behavior

**DOI:** 10.1371/journal.pone.0040121

**Published:** 2012-07-06

**Authors:** Venkat Padmanabhan, Zeina S. Khan, Deepak E. Solomon, Andrew Armstrong, Kendra P. Rumbaugh, Siva A. Vanapalli, Jerzy Blawzdziewicz

**Affiliations:** 1 Venkat Padmanabhan Department of Mechanical Engineering, Texas Tech University, Lubbock, Texas, United States of America; 2 Zeina S. Khan Department of Chemical Engineering, Texas Tech University, Lubbock, Texas, United States of America; 3 Deepak E. Solomon Department of Chemical Engineering, Texas Tech University, Lubbock, Texas, United States of America; 4 Andrew Armstrong Department of Surgery, Texas Tech University Health Sciences Center, Lubbock, Texas, United States of America; 5 Kendra P. Rumbaugh Department of Surgery, Texas Tech University Health Sciences Center, Lubbock, Texas, United States of America; 6 Siva A. Vanapalli Department of Chemical Engineering, Texas Tech University, Lubbock, Texas, United States of America; 7 Jerzy Blawzdziewicz Department of Mechanical Engineering, Texas Tech University, Lubbock, Texas, United States of America; University of Zurich, Switzerland

## Abstract

*Caenorhabditis elegans*, a free-living soil nematode, displays a rich variety of body shapes and trajectories during its undulatory locomotion in complex environments. Here we show that the individual body postures and entire trails of *C. elegans* have a simple analytical description in curvature representation. Our model is based on the assumption that the curvature wave is generated in the head segment of the worm body and propagates backwards. We have found that a simple harmonic function for the curvature can capture multiple worm shapes during the undulatory movement. The worm body trajectories can be well represented in terms of piecewise sinusoidal curvature with abrupt changes in amplitude, wavevector, and phase.

## Introduction

### Motivation

A one-millimeter long soil-dwelling nematode *C. elegans* is a model organism for comprehensive investigations spanning genetics [1], [2], neural control [3], [4], sensory transduction [5], behavior [6], [7] and locomotion [8], [9]. With a total of 959 nongonadal cells, of which exactly 302 are neurons [3], *C. elegans* is a very simple organism. Yet, it can efficiently move in complex environments [10]–[14], purposefully adjust its behavior to mechanical [15]–[17], chemical [18], [19], and thermal stimuli [20]–[24], and learn [20], [25].

So far many studies of nematode behavior have focused on gross characteristics, such as the probability of turns or the fraction of worms that approach a chemical signal within a prescribed time [25]–[27]. Also, typical body postures of *C. elegans* (e.g., sinusoidal undulations, shallow turns, and sharp 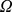
- or loop-turns) have been classified and used to characterize worm trajectories. Such descriptions of worm motion, however, are inadequate because they do not address more advanced questions regarding worm behavior: for example, how the nervous, sensory, and motor systems of *C. elegans* control its body to generate efficient propulsion and move the nematode towards a food source or away from danger. To gain insight into such complex behaviors a quantitative approach to model worm dynamics is a prerequisite.

An important contribution to develop quantitative models of worm gait has been made by Stephens *et al.* [28]. They have shown that a set of complex shapes that *C. elegans* assumes during crawling has a simple representation in orientational coordinates intrinsic to the worm body. In their model a significant fraction of such shapes are expressed in terms of several eigenmodes of a correlation matrix for orientation of body segments. This “eigenworm” description provides a low dimensional representation of the complex space of worm postures, which is a crucial step towards developing realistic models of the worm behavior.

On the other hand, the eigenworm picture [28] is not intuitive, and it misses an important characteristic of worm trajectories, recently described by Kim *et al.* [29]. Based on an analysis of trajectory images, they have proposed [29] that shallow turns of crawling worms can be described using a model where the worm performs simple sinusoidal undulations with suddenly changing parameters such as wavelength and amplitude. While this model agrees well with experimental observations of *C. elegans* performing low-amplitude undulations [29], it is not sufficient for a description of 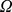-turns and other sharp turns.

### Piecewise-harmonic-curvature Model

Our curvature-based description of *C. elegans* motion is motivated by geometrical observations of trails of nematodes moving without slip on a soft agar substrate. In this work we build on the ideas of [28] and [29] to develop a simple curvature-based representation of worm kinematics, obtaining a model that overcomes the shortcomings mentioned above. As in [29], we analyze entire trails of crawling nematodes (rather than their individual body shapes) to capture sudden changes of the gait that the worm uses to navigate its environment. However, we formulate our description in terms of intrinsic geometric quantities (arc length 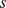 and local curvature 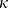), rather than real-space coordinates, and this allows us to go beyond describing only shallow turns and low-amplitude undulations. As a result, we obtain a low-dimensional representation of all worm postures and trajectories in terms of uncomplicated basis functions.

**Figure 1:**
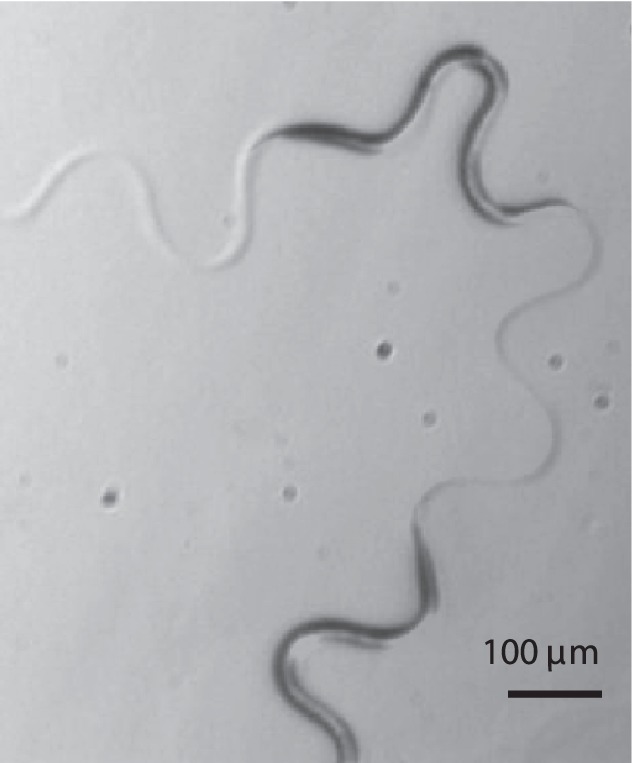
Trail and body postures of *C. elegans* crawling on agar. The figure shows two superimposed snapshots of the nematode crawling on a 4 *wt*% agar surface.

A typical trail is shown in Fig. 1, where two images are superimposed to depict the worm postures at different times. The worm makes deep U-shaped undulations throughout the trajectory. The depth of the undulations varies in time, and due to these variations the overall direction of the worm motion changes. The worm occasionally assumes an 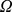-like shape (as seen in the top right corner of Fig. 1), which results in a significant change of direction.

#### Assumptions

Our piecewise-harmonic-curvature (PHC) model is built on the following three assumptions: (i) the worm controls its body posture by propagating the curvature backwards along the body, (ii) the curvature wave is generated in the front (head) segment of the worm body; the time variation of the curvature of this segment serves as the boundary condition for the curvature wave and determines this wave completely, and (iii) the variation of the head segment curvature can be described in terms of a simple piecewise-harmonic function.

As shown below, our model is confirmed by a detailed analysis of trails of worms crawling without slip on agar substrate. Trail segments described by harmonic variation of the curvature are clearly identified, and the jumps of the curvature-wave amplitude, wavevector, and phase from segment to segment are determined. We thus obtain an analytic representation of the entire trail in terms of a relatively small number of parameters.

## Results

### Curvature Representation of the Worm Trail

**Figure 2:**
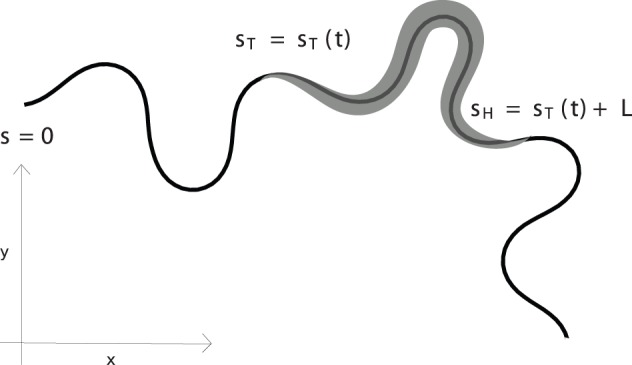
Schematic of a *C. elegans* trail. The worm body is a portion of the entire trajectory.

It is convenient to describe nematode trails and instantaneous body shapes using the parametric description
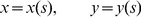
(1)for the Cartesian 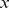 and 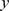 coordinates of all points along the trail. The parameter 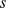 in Eq. (1) represents the arc length along the trail. The worm body posture at time 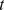 corresponds to a portion of the trail between the points
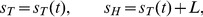
(2)where 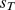 is the tail position, 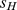 is the head position, and 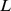 is the length of the worm. If the worm is moving with a constant velocity 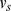, the tail position is given by
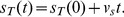
(3)The above description of the trail geometry is illustrated in Fig. 2.

The shape of the worm trail in the parametric form (1) can be evaluated from the local curvature 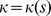 using the Frenet–Serret equations
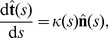
(4)where
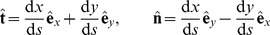
(5)are the unit vectors tangent and normal to the trail, respectively. Combining relations (4) and (5) yields a set of coupled second-order differential equations



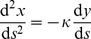
(6a)

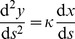
(6b)for the trail shape (1). Equations (6) are solved with the initial conditions



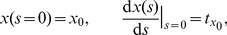
(7a)

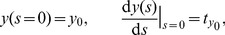
(7b)where 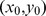 is the position of the worm tail at the beginning of the trail and the tangent unit vector, 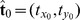 describes the orientation of the tail at 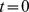.

### Harmonic-curvature Variation

We argue that worm trails (and the corresponding sequences of worm shapes) can be accurately described in terms of a simple harmonic-curvature variation
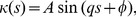
(8)where 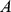 is the amplitude, 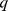 is the wavevector, and 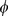 is the phase of the harmonic wave. Below we demonstrate that a combination of segments of the lines obtained by integration of the curvature (8) with different values of the parameters 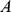, 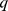, and 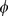 can be used to construct the entire worm trail.

**Figure 3:**
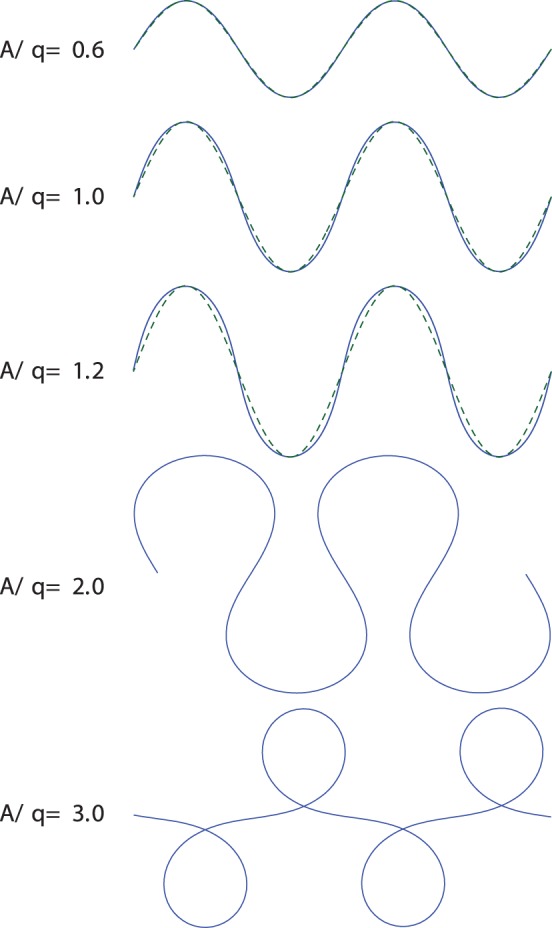
Family of shapes obtained from a harmonic curvature function. Solid lines show the solutions of Frenet–Serret equations (6) with the harmonic curvature (8) with different values of normalized amplitude 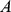/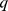. The dashed lines correspond to the real-space sinusoidal form (9).

**Figure 4:**
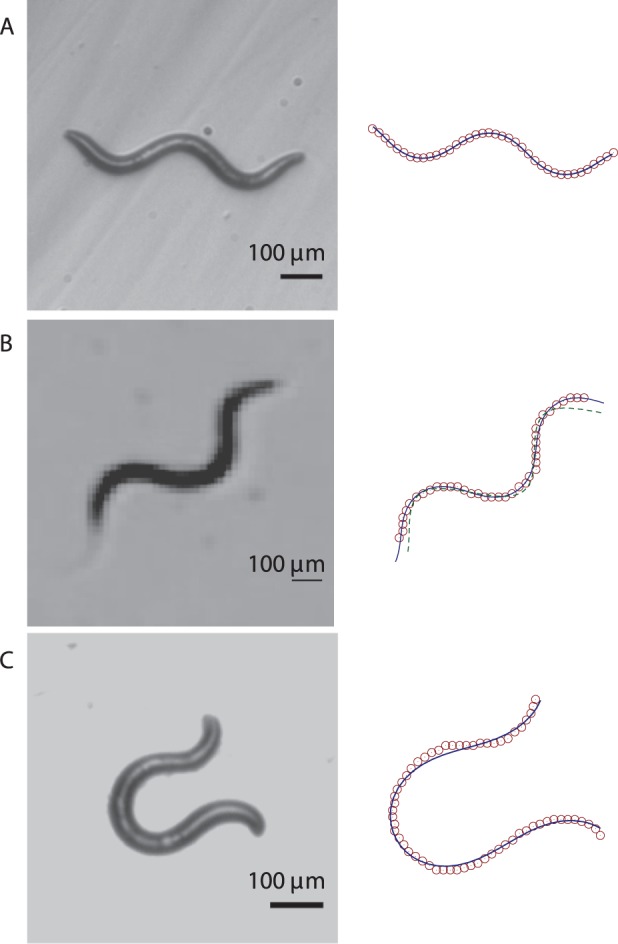
Body postures of *C. elegans* crawling on agar. Left column: Experimental images. Right column: corresponding fits of the harmonic-curvature representation (8) (solid lines) to the skeletonized data (open circles). For clarity, only every fifth point of the skeleton data is plotted in all the figures. Figure (B) also shows the real-space sinusoidal fit (9) (dashed line), which significantly deviates from the skeleton data.

**Figure 5:**
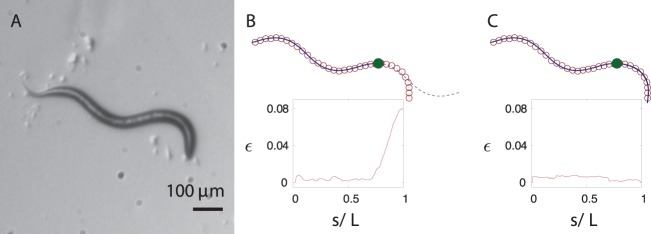
Body posture of *C. elegans* with two distinct piecewise-curvature modes. (A) The experimental image of the worm. (B) The skeleton data (open circles) and the harmonic-curvature fit for the tail segment (solid line). The extension of the fit that does not follow the head segment is shown by dashed line, and the point where the piecewise-curvature mode changes is indicated by a filled circle. (C) The best fit of the two-mode harmonic-curvature representation (8) (solid line). Since the line is obtained by integrating second-order differential equations (6), it is continuous and has a continuous slope. The insets in (B) and (C) show the local fit error (17) along the skeleton of the nematode. For the single-curvature-mode fit (B) the error rapidly increases after the point indicated by the filled circle, whereas for the continuous two-mode fit (C), the local error is below 1% along the whole body.

**Figure 6:**
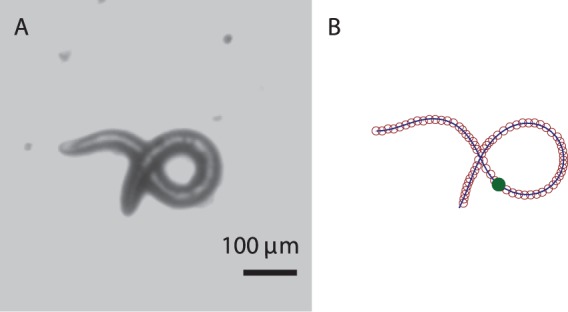
Loop turn with two distinct piecewise curvature modes. (A) Experimental image. (B) The two-mode harmonic-curvature representation (solid line); the skeleton data (open circles); the location of the piecewise-mode change (filled circle).

**Figure 7:**
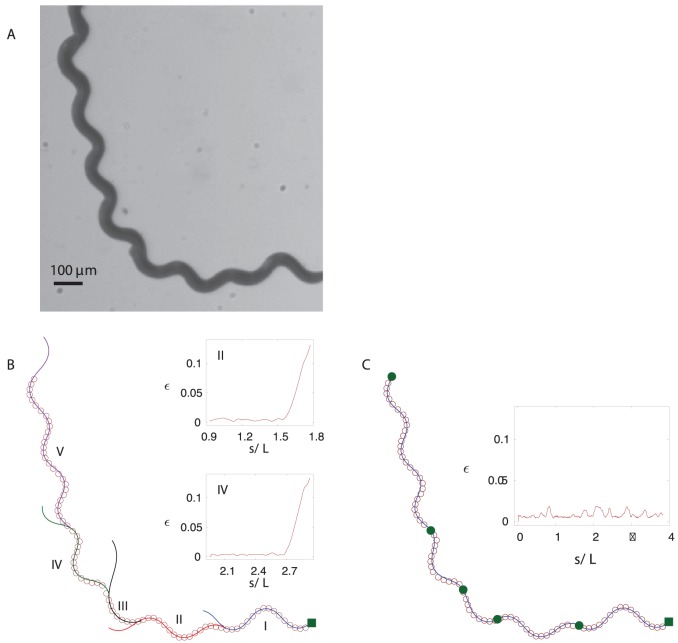
Piecewise-harmonic-curvature (PHC) representation of a *C. elegans* trail. (A) Worm trail obtained by superposing individual video frames. (B) Independent single-mode harmonic-curvature fits (solid lines) to different regions of the skeleton data (open circles). Different segments are shown in different colors, and the filled square marks the beginning of the trail. Sudden deviations of the single-mode fits from the skeleton data indicate that the worm switched its piecewise-mode. The insets show the local fit error (17) for the second and fourth trail segment. (C) A continuous PHC representation obtained by fitting the solution of equations (6) and (11) to the trail-skeleton points. The end of each piecewise-mode is marked with a solid circle. The inset shows the local fit error.

**Figure 8:**
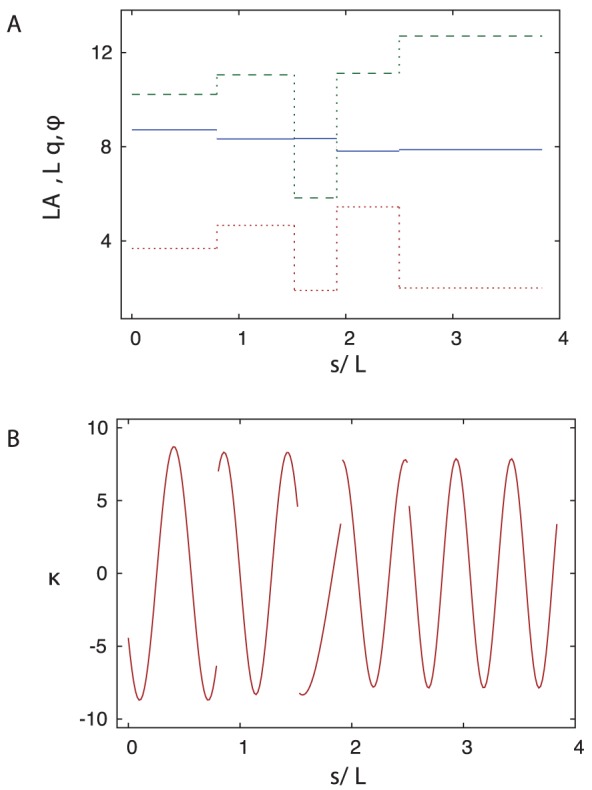
Parameters of the PHC representation. (A) The normalized amplitude 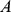 (blue – solid), wavevector 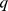 (green – dashed), and phase 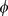 (red – dotted) of the curvature wave along the trail shown in Fig. 7. (B) The evolution of curvature along the same trail.

**Figure 9:**
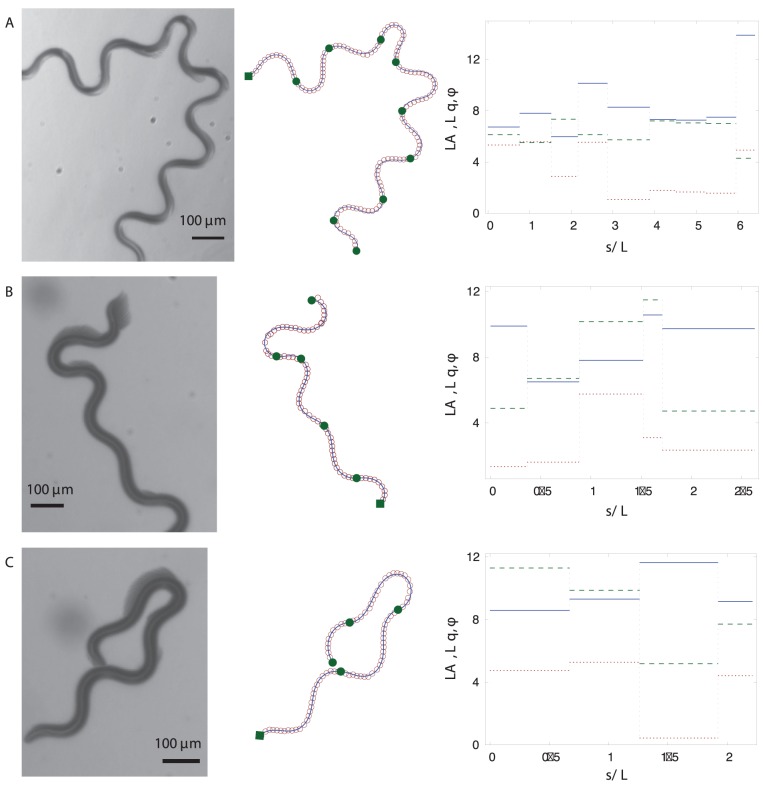
PHC representations of trails and the corresponding piecewise-curvature mode parameters. Left column: experimental images of several trails of *C. elegans*. Middle column: the PHC representations (11) (solid lines) of the skeletonized trail data (open circles). The beginning of the whole trail is marked with a solid square; the end of each piecewise-curvature mode is marked with a solid circle. Right column: The normalized amplitude 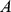 (blue – solid), wavevector 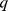 (green – dashed), and phase 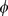 (red – dotted) of the piecewise-curvature modes along the trail.

**Figure 10:**
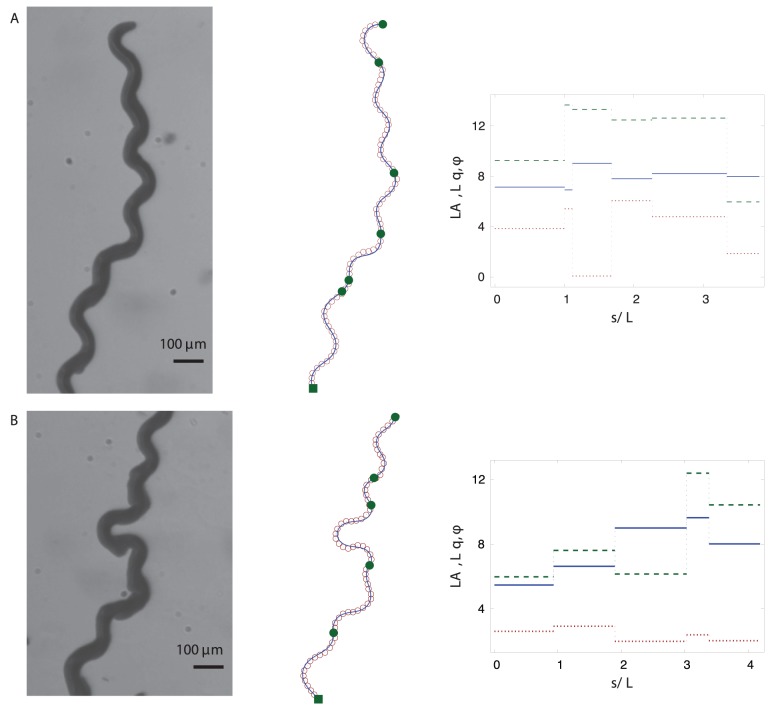
PHC representations and the corresponding piecewise-curvature mode parameters for additional trails.

The family of shapes obtained by changing the amplitude 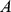 in Eq. (8), with 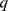 and 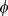 fixed, is depicted in Fig. 3. Other shapes, corresponding to different values of 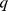 and 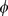, can be obtained from this family by affine transformations. A comparison of the lines plotted in Fig. 3 with the worm tracks and body shapes shown in Figs. 1 and 4, 5, 6, 7, 8, 9, and 10 reveals close similarity. For example, the shape of the line with the dimensionless amplitude 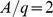 closely resembles the 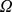-turns made by the worm in Figs. 4C and 9. Similarly, for 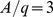, the shape resembles the loop turn seen in Fig. 6.

In Fig. 3 the curvature-based harmonic-wave description (8) is compared with the real-space harmonic wave
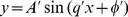
(9)that was used in [29] to describe worm trails with shallow turns. For low amplitudes of the curvature wave 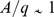 the two relations (8) and (9) yield nearly equivalent shapes, but the results significantly differ outside this range. An analysis of the skeletonized worm shapes depicted in Fig. 4 shows that our harmonic-curvature model (8) describes nematode body postures accurately for all amplitudes. The real-space harmonic description (9) becomes inaccurate already for moderate-amplitude undulation (cf. Fig. 4B).

It is also worth noting that there is a striking similarity between the curves followed by crawling *C. elegans* and the trajectories of bacterial pathogen *Listeria monocytogens* [30]. In both cases, the organisms move along lines described by the harmonic curvature variation. However, in the case of *L. monocytogens* the parameters of the harmonic curvature wave vary gradually along the trajectory, whereas *C. elegans* change these parameters discontinuously, as described below.

### Piecewise-harmonic-curvature Function

Our simple harmonic-curvature model (8) can be applied to describe both individual worm postures and entire nematode trails (such as the trail depicted in Fig. 1). Complete trails that include gradual and deep turns can be represented with high accuracy using a piecewise-harmonic-curvature function. We first discuss piecewise-harmonic representation for individual nematode postures, and then we analyze trajectories of crawling worms.

#### Worm body postures

The shape of *C. elegans* shown in Fig. 5 has a distinctly higher curvature in the head section than in the tail section. For such a shape, a single-mode sinusoidal-curvature representation is insufficient. Figure 5B shows a line obtained by integrating Frenet–Serret equations (6) with the harmonic curvature (8) and parameters adjusted to fit the tail part of the worm. From the tip of the tail upto the point indicated by the filled circle, the line matches the skeletonized nematode posture very well. Beyond this point a sudden deviation occurs, as determined by the local error shown in the inset. Therefore, another harmonic mode is necessary to describe the next segment of the worm. It follows that the worm exhibits more than one curvature mode along its body length, with an abrupt transition between the modes.

To accurately describe the entire worm posture, we use the piecewise-harmonic curvature
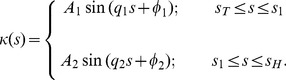
(10)Here 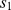 is the intermediate point at which a sudden transition between the two harmonic modes occurs. As demonstrated in Fig. 5C, the two-mode curvature function (10) yields an accurate description of the shape of the whole worm from tail to head.

Two-segment harmonic fits of similar quality have been obtained for other nematode shapes, such as the loop posture depicted in Fig. 6. At the switching point 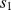 the worm curvature is often discontinuous, but the worm contour obtained via integration of the second-order Frenet–Serret differential equation (6) is continuous and has a continuous orientation.

#### Worm trails

For *C. elegans* crawling without slip on a solid surface, the instantaneous body postures can be treated as segments of the entire worm trail (cf. Fig. 1). Therefore, we assume that the abrupt changes in the parameters of the curvature wave that propagates throughout the worm body are reflected in the overall trail shape.

A typical example of a trail made by *C. elegans* crawling on an agar surface is depicted in Fig. 7A. The picture was obtained by superimposing consecutive frames of a video recording of a crawling worm, as described in the Methods section. The worm undergoes a small amount of slip, which is reflected in occasional trail imperfections. These imperfections are also due to the random movement that the head of the nematode makes before deciding on the path of its motion. However, once the head continues to move in a certain direction, the rest of the body follows the head on a determined path.

The trail of a gradually turning worm shown in Fig. 7A cannot be represented using a single harmonic-curvature mode. However, similar to the results for worm postures (cf. Fig. 5), individual segments of the trail are consistent with the harmonic-curvature representation, as illustrated in Fig. 7B. In a certain range of 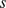 each local harmonic-curvature fit follows the track with high accuracy, and then rapidly deviates from the track as shown in the insets in Fig. 7B. The errors of fits are less than 1% up to the piecewise-mode transition point and then show a steep increase.

Based on the above observations, we expect that the entire trail can be well represented by a continuous line corresponding to the piecewise-harmonic curvature
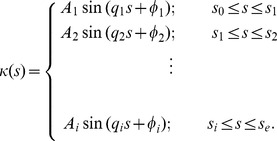
(11)Here 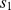, 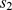, … 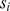 denote the intermediate points of the worm’s trail where a sudden transition between harmonic modes occurs, and 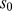 and 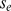 are the beginning and end points of the trail, respectively. The continuous PHC representation for the whole trail is depicted in Fig. 7C. The inset in Fig. 7C shows the local deviation of our PHC model from the skeleton data. The low deviation (less than 1% with occasional jumps to about 2%) for all values of the arc-length parameter 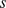 indicates that our model fits the data with high accuracy.

Figure 8A shows the discontinuous variation in the curvature-wave amplitude, wavevector and phase along the worm trail depicted in Fig. 7A. Jumps of these three parameters occur at irregular intervals, and the size of the jumps is also irregular. The corresponding evolution of the curvature is illustrated in Fig. 8B. The curvature is discontinuous at each piecewise-mode shift, but the final real-space PHC representation, obtained via integration of the second order differential equation (6), is continuous.

Further examples of worm trails and the corresponding PHC representations are shown in Figs. 9 and 10. Our description captures well both the gradual changes of the overall path direction (cf. Figs. 7 and 10) and rapid changes such as 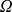-turns and other deep turns (cf. Fig. 9). An analysis of the variation in the curvature-wave parameters indicates that a deep turn may result from an increase of the wave amplitude, a decrease of the wave vector, or from both such changes occurring at the same time.

The nematode *C. elegans* changes suddenly its undulation mode not only during forward motion, but also in the backward crawling gait. It has been observed [31] that upon reversal of its direction of motion *C. elegans* often retraces part of its track, and then abruptly veers from the previous path. This deviation has a very similar geometry to the abrupt divergence between the trail of a worm crawling forward and a single-piecewise-mode fit to the trail (cf. Fig. 7B). We conclude that both deviations result from the same general behavioral pattern, i.e., from a sudden change in parameters of the harmonic-curvature wave propagating through the body of *C. elegans*.

## Discussion

Our piecewise-harmonic-curvature model provides a simple analytical description of the crawling motion of *C. elegans* in terms of elementary sinusoidal movements in curvature space. We have demonstrated that the worm turns by changing the curvature-wave parameters. In this section we describe consequences of our findings and indicate directions for further research.

### Curvature Evolution in the Intrinsic Worm Coordinates

Our observations provide important clues on how the neural system of *C. elegans* controls the nematode movement to generate propulsion and navigate the environment. To elucidate this aspect, we transform our curvature-based description to the intrinsic worm coordinates with the arc length 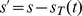 measured from the position of the tip of the nematode tail, instead of the beginning of the trail. In this representation, the curvature 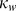 at position 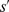 and time 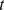 can be expressed in the form
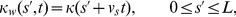
(12)where 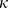 is the piecewise-harmonic function (11). To describe the tail position 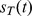 we used Eq. (3), on the simplifying assumption that the worm moves along the trail with a constant velocity 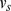. (According to our observations, the velocity 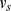 often stays constant over several harmonic-curvature segments of the trajectory, and it undergoes occasional jumps at the curvature-mode transition points. Incorporation of such jumps into Eqs. (12)–(15) is straightforward. In some cases we observed significant velocity changes over the length of a single mode, usually at the portions of the trail where the PHC model shows an increased fitting error. The large error and varying velocity indicate a deviation from the stereotyped behavior captured by our model.)

#### The worm’s point of view on piecewise curvature modes

According to our model, the worm motion is entirely determined by the initial worm configuration at 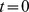,
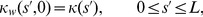
(13)and the boundary condition
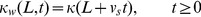
(14)that describes the head curvature at time 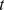. The curvature generated in the head segment propagates along the worm body according to relation (12).

It follows that the worm (i.e., the nervous system that controls its body movements) needs to perform three, relatively simple, independent tasks:

(i) generate a harmonic oscillation of the curvature in the head part of the body, corresponding to the relation
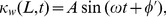
(15)where 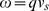 is the angular frequency;

(ii) propagate the curvature wave backwards along the nematode, as described by Eq. (12);

(iii) produce random jumps of the amplitude 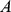, wavevector 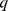, and phase 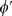 of the harmonic time variation in the head curvature (15).

Since the curvature is an intrinsically local quantity, all three tasks require only local operations. Generation of the curvature in the head segment (tasks (i) and (iii)) involves local muscle contractions. Propagation of the curvature wave (task (ii)) depends on local muscle contractions and communication between neighboring segments.

The simplicity of our geometrical description strongly suggests that there is an underlying simplicity in the way *C. elegans* controls its motion. The worm is not aware of its entire posture, which would require long-range communication between spatially separated body segments, so the nematode controls its body locally. The above conjecture is also consistent with results of a recent study by Stirman *et al.* [32]. By targeted illumination, these researchers have manipulated the behavior of optogenetically engineered *C. elegans*. In one of their experiments (cf., Supplementary Video 1 of [32]) neural excitation produced by a laser-light impulse applied to the head section of a crawling nematode created a sharp bend in the worm body. After this initial perturbation, the bend propagated backwards along the body of *C. elegans*, causing the nematode to follow the direction of the head, and resulting in a worm crawling along an artificially predetermined path (in the shape of a triangle). This behavior supports our conclusion that the propagation of the curvature wave along the worm body is independent of the curvature-generation mechanism.

### Worms Moving in Different Environments

In this paper we have focused on 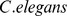 moving without slip on a solid substrate. However, based on our discussion of the way the worm controls its body movements, we expect that key elements of our description are likely to apply more generally, i.e., even when *C. elegans* undergoes a significant slip, and the nematode trail is not so well defined.

A typical example of such a situation is *C. elegans* swimming in water. During swimming, the nematode undergoes a significant translational and rotational drift with respect to the surrounding fluid. Thus a superposition of subsequent nematode images does not show a well-defined trail. To describe the motion of *C. elegans* in its swimming gait, it is more convenient to analyze a sequence of individual images (rather than their superposition) using intrinsic worm coordinates, i.e., the framework defined by equation (12).

**Figure 11:**
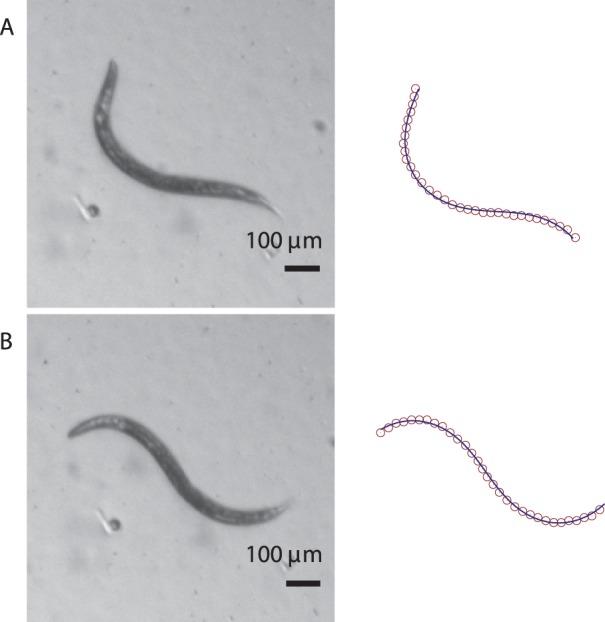
Body postures of *C. elegans* swimming in water. (A) 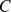-shaped and (B) sine-shaped nematode postures. Left column: experimental images. Right column: the corresponding skeletonized data and single-mode harmonic-curvature fits.

**Figure 12:**
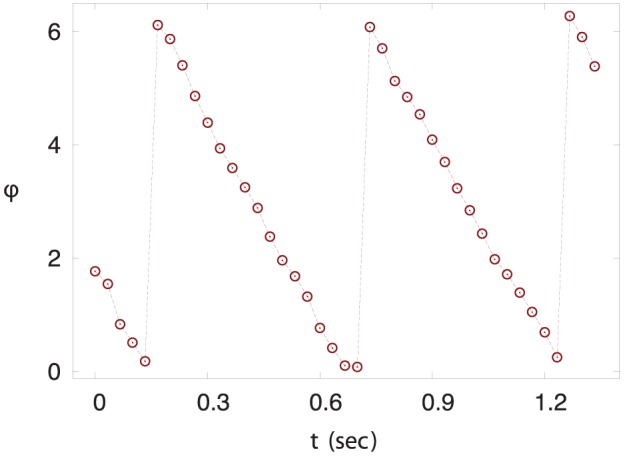
The phase evolution 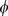
 **for a sequence of images of** ***C. elegans*** **swimming in water.** The sequence of images from the movie of a swimming *C. elegans* was fitted using harmonic-curvature function (8) with the phase 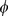 varying from frame to frame and the amplitude 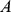 and wavevector 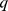 identical for all frames.

Two images from a video of a swimming *C. elegans* are shown in Fig. 11 together with the harmonic-curvature fits to the numerical skeletonization data. The nematode posture in each frame can be accurately represented by contours corresponding to the harmonic curvature (8) with a single curvature mode of the same amplitude and wavevector 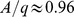 and 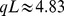, but a varying phase 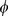. This suggests that the internal kinematics of a swimming and crawling *C. elegans* are quite similar. The evolution of phase 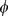 as a function of time for a sequence of video images is shown in Fig. 12. The gradual drop in 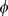 with constant 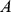 and 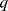 suggests that a single wave propagates backward as the nematode swims.

A more detailed analysis is needed to determine whether *C. elegans* in the swimming gait uses a similar strategy to turn as in the crawling gait. However, based on previous reports that the worm uses 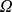-turns for changing swimming direction [33], we postulate that a swimming worm, similar to a crawling worm, abruptly varies the harmonic-wave parameters at irregular intervals, which results in a modification of the swimming course.

We believe that the framework based on harmonic-curvature-wave assumptions (11) and (12) will be useful in the analysis of worm propulsion and maneuverability in water. We also expect that a similar framework can be applied in microstructured environments, such as soil or arrays of microfluidic pillars.

### Conclusions and Future Directions

Our study has demonstrated a significant geometrical simplicity of the crawling gait of *C. elegans*. We have shown that *C. elegans* propels itself using a simple set of elementary movements, and that both shallow turns and sharp loop- and 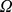-turns correspond to the same geometrical family of contours generated by a harmonic variation of the curvature along the body of the nematode. The geometrical simplicity revealed by our analysis directly reflects key underlying aspects of the neural control of the nematode motion (as explained above).

The PHC description of the nematode kinematics provides a powerful tool for quantitative investigations of the worm behavior. For example, behavioral changes of *C. elegans* in response to chemical signals [7], [19], [34] can be quantified by the statistics of jumps of the curvature-wave parameters 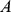, 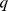, and 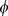. Since the worm controls these parameters directly (in contrast to the angles of the corresponding turns), further studies based on the PHC model are likely to reveal important information on underlying mechanisms of chemotaxis. In particular, we will show in a forthcoming publication that the PHC model enables a unified description of the pirouette [7] and gradual-turn [34] strategies that *C. elegans* uses to navigate towards a chemoattractant.

We expect that our analysis will facilitate development of advanced models of neural control of nematode motion. Current models [35] capture gross qualitative features of the crawling gait of *C. elegans* fairly well. However, as implied by our results, more comprehensive models should include separate neural-network functional units that would (i) control curvature-wave generation in the head segment of the worm, (ii) propagate the wave along the worm body, and (iii) change the wave parameters. Introduction of such models would be an important step towards back-engineering of the neural control system of *C. elegans*. Such models would also aid development of a control system for future synthetic worm-like particles that can crawl autonomously.

## Materials and Methods

### Experimental Details

#### Worm preparation

*C. elegans* were cultured on a 60
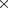
15 mm petri dish containing 4 *wt*% agar at 20°C. The worms were fed every 4 days with 0.05 ml of E. coli, and the petri dish was subsequently wrapped with paraffin film to prevent the growth of other bacteria or mold. To conduct experiments, a chunk of agar containing worms was transferred onto a fresh bacteria-free 4 *wt*% agar plate [36]. Images were acquired once all the worms had left the chunk and had begun crawling on the fresh plate.

#### Image acquisition

Images of *C. elegans* crawling on agar plates were taken using a Zeiss Stemi 2000-C stereo microscope with a StreamView CCD camera (640
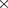
480 pixels with a 7.4 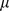
m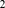 pixel size and an 8-bit mono sensor), typically acquiring between 10–30 frames per second, and were saved as black and white avi movie files. The agar plates were illuminated from below by mounting the plates on one face of a right angle prism with an aluminized hypotenuse (50 mm leg length, 70.7 mm hypotenuse length). A Chiu Technical Corporation Lumina FO-150 fiber optic illuminator with a focusing lens and light diffuser were used to illuminate one right-angle face of the prism; the light was directed through the sample by the hypotenuse face of the prism, and was acquired by the stereo microscope. Worms imaged with this apparatus appear as a dark feature on a light background.

#### Image analysis

Individual worm shapes were obtained from the avi movie files by converting the movie to 8-bit black and white tiff images using ImageJ software (http://rsbweb.nih.gov/ij/). The individual images were then analyzed as follows. A greyscale threshold was applied to the images to separate the worm from the background, small non-worm objects were removed from the images based on their size, ‘holes’ in the thresholded worms’ bodies were filled and the solid binary images of the worms’ bodies were transformed into a skeleton running down the center of the body by applying a morphological thinning operation. These operations were carried out using the Matlab Image Processing toolbox routines *bwareaopen.m*, *imfill.m*, and *bwmorph.m* respectively. Individual data points were collected from the resulting binary skeleton image by determining which pixels had a value equal to 1, collecting their row and column positions, and utilizing these positions as Cartesian 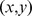 coordinates in order to perform curvature fits. In the case of Fig. 6, the above described method of automatic skeletonization using the image processing software did not provide analyzable skeleton data due to the overlapping body segments of the worm. Therefore, in this case, the image processing software was used to obtain the skeleton of the worm except in the overlapping region. For the overlapping region, the position of the pixels along the centroid of the worm were selected and joined with the rest of the worm body.

Worm trajectories, such as those shown in Figs. 7, 9, and 10, were constructed by opening an avi movie of crawling worms in ImageJ, and projecting the darkest pixels from the time-series of images into a single image (using ImageJ’s *Z project* tool). This resulting image was then processed in the same manner as individual worm images in order to obtain 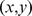 data points along the worms’ trajectories.

### Modeling Details

#### Solution of differential equations

The set of coupled differential equations (6) was solved using the Matlab’s fourth-order Runge-Kutta ODE solver *ode45.m*. Since Eq. (6) is a set of second order differential equations, it requires two initial conditions––the starting point and the initial direction. These two conditions were obtained by the fitting procedure described below. For the continuous representation with multiple piecewise-modes along the trajectory, each mode was determined using the location and direction of the last point of the previous mode as the initial conditions. With sudden jumps in parameters for the harmonic-curvature function, the curvature is discontinuous between each mode as shown in Fig. 8B. However, the corresponding PHC representation of the trajectory in real (Cartesian) space is a continuous curve with a continuous slope, because it is a solution of a set of second-order differential equations.

#### Measure of error

The deviation of the model from the experimental data was measured using the error function
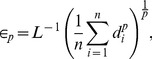
(16)where 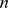 is the number of data points, and 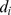 is the distance of the curve (1) from the data point 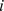. The quantity (16) was minimized to obtain the model parameters for accurate description of the worm body postures and trajectories. In all cases except one we used 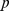 = 2. For the trail presented in Fig. 9A, we used 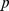 = 12 to avoid large local deviations between the model and the data. The error (16) was minimized using the built-in Matlab optimizer *fminsearch.m*.

The local errors
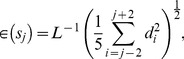
(17)shown in the insets of Figs. 5 and 7 were estimated by calculating the average distance of the curve from the data along a moving window of five data points.

### Model Parameters 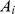, 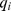, and 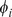


The model parameters for a given trajectory were evaluated in two stages. In the first stage, individual segments of the trajectory were fitted independently using the single curvature-mode representation (8) to find the positions of the piecewise-mode shifts and to determine the initial guesses for the parameters 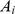, 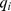, and 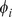. In the second stage, a continuous multi-mode representation of the trajectory was obtained using the initial guesses from the first step.

#### Single-mode independent piecewise fits

The fitting procedure was started from one end of the trail with approximately 25 consecutive skeleton points. The model parameters for this section were obtained by minimizing Eq. (16). The quality of the fit was estimated by calculating the local deviation between the model and the experimental data using Eq. (17). If the local error of a particular segment showed no increase, the length of the section was doubled and the above steps were repeated. If the local error showed a steady increase beyond a certain point, as seen in Fig. 7B, the fit was truncated at that point and the point of truncation served as the location of a mode shift. The above steps of independent harmonic fits were repeated until the end of the trajectory was reached.

#### Continuous fit

The purpose of this stage was to determine the parameters 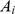, 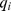, and 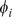 for the continuous representation of a trajectory according to the PHC model (11). The continuous representation was obtained by consecutively fitting larger and larger portions of the trajectory to avoid difficulties associated with local minima of the accuracy measure (17). The fitting procedure was started from the first segment and involved addition of subsequent segments in two steps. In the first step, the curvature parameters of segment 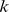 were refitted with the constraints of maintaining the continuity of the curve and its slope between the current and previous segments. In the second step, segments 1 through 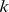 were readjusted allowing variations of their curvature parameters and locations of piecewise-mode shifts. Typically, we allow only variations of parameters of segments 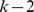, 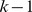, and 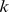 in this step. The above two steps were repeated until the end of the trajectory was reached.

In several cases (Figs. 7, 9B, and 10A), the above procedure did not provide an accurate match between the model and the trajectory. This difficulty was associated with defects of the trail due to the local slip of the worm. For these cases, the continuous PHC representation of the trajectories was obtained by first fitting the two sections (before and after the region with imperfections) independently, and then joining them smoothly using an additional segment corresponding to the region of the imperfect data. For all trajectories, the accuracy 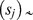 0.02 was achieved, and the typical segment length was 0.7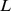.

To verify the robustness of our PHC model, we carried out additional calculations in which the sequential fitting process was performed in the opposite direction, i.e., starting from the trail end. In spite of a different starting point and different order of segment reconstruction, the forward and backward fits produced equivalent results. Similar values of the PHC parameters were also obtained by fitting individual worm body postures rather than the entire trail. The above tests, as well as the rapid increase of the single-mode fit error beyond the mode-switch point (as illustrated in Figs. 5 and 7), indicate that the PHC model is robust and faithfully reflects the nematode behavior.

## References

[1] Fire A, Xu SQ, Montgomery MK, Kostas SA, Driver SE (1998). Potent and specific genetic interference by double-stranded RNA in Caenorhabditis elegans.. Nature.

[2] Grishok A, Tabara H, Mello CC (2000). Genetic requirements for inheritance of RNAi in C. elegans.. Science.

[3] White JG, Southgate E, Thomson JN, Brenner S (1986). The Structure of the Nervous System of the Nematode Caenorhabditis elegans.. Philos Trans R Soc London, Ser B.

[4] Chen B, Hall D, Chklovskii D (1986). Wiring optimization can relate neuronal structure and function.. Proc Natl Acad Sci USA.

[5] Bargmann CI, Kaplan JM (1998). Signal transduction in the Caenorhabditis elegans nervous system.. Ann Rev of Neurosci.

[6] Yen CF, Wyart M, Xie J, Kawai R, Kodger T (2010). Biomechanical analysis of gait adaptation in the nematode Caenorhabditis elegans.. Proc Natl Acad Sci USA.

[7] Pierce-Shimomura JT, Morse TM, Lockery SR (1999). The fundamental role of pirouettes in Caenorhabditis elegans chemotaxis.. J Neurosci.

[8] Grey J, Lissmann HW (1964). The Locomotion of Nematodes.. J Exp Biol.

[9] Karbowski J, Cronin JC, Seah A, Mendel JE, Clearya D (2006). Conservation rules, their breakdown, and optimality in Caenorhabditis sinusoidal locomotion.. J Theo Biol.

[10] Sawin ER, Ranganathan R, Horvitz HR (2000). C-elegans locomotory rate is modulated by the environment through a dopaminergic pathway and by experience through a serotonergic pathway.. Neuron.

[11] Qin J, Wheeler AR (2007). Maze exploration and learning in C-elegans.. Lab on a Chip.

[12] Park S, Hwang H, Nam SW, Martinez F, Austin RH (2008). Enhanced Caenorhabditis elegans Locomotion in a Structured Microuidic Environment.. PLoS One.

[13] Jung S (2010). Caenorhabditis elegans swimming in a saturated particulate system.. Phys Fluids.

[14] Majmudar T, Keaveny EE, Zhang J, Shelley MJ (2012). Experiments and Theory of Undulatory Locomotion in a Simple Structured Medium.. J R Soc Interface.

[15] Shen XN, Arratia PE (2011). Undulatory Swimming in Viscoelastic Fluids.. Physical Review Letters 106.

[16] Sznitman J, Shen X, Purohit PK, Arratia PE (2010). The Effects of Fluid Viscosity on the Kinematics and Material Properties of C. elegans Swimming at Low Reynolds Number.. Experimental Mechanics.

[17] Berri S, Boyle JH, Tassieri M, Hope IA, Cohen N (2009). Forward locomotion of the nematode C. elegans is achieved through modulation of a single gait.. HFSP Journal.

[18] Faumont S, Miller AC, Lockery SR (2005). Chemosensory behavior of semi-restrained Caenorhabditis elegans.. Journal of Neurobiology.

[19] Pierce SJT, Dores M, Lockery SR (2005). Analysis of the effects of turning bias on chemotaxis in C. elegans.. Journal of Experimental Biology.

[20] Goodman MB, Schwarz EM (2003). Transducing touch in Caenorhabditis elegans.. Ann Rev of Physio.

[21] Chalfie M, Sulston JE, White JG, Southgate E, Thomson JN (1985). The neural circuit for touch sensitivity in Caenorhabditis elegans.. J Neurosci.

[22] Matsuoka T, Gomi S, Shingai R (2008). Simulation of C. elegans thermotactic behavior in a linear thermal gradient using a simple phenomenological motility model.. J Theo Biol.

[23] Ramot D, MacInnis BL, Lee HC, Goodman MB (2008). Thermotaxis is a Robust Mechanism for Thermoregulation in Caenorhabditis elegans Nematodes.. J Neurosci.

[24] Nakazato K, Mochizuki A (2009). Steepness of thermal gradient is essential to obtain a unified view of thermotaxis in C. elegans.. J Theo Biol.

[25] Beale E, Li G, Tan MW, Rumbaugh KP (2006). Caenorhabditis elegans senses bacterial autoinducers.. Applied and environmental microbiology.

[26] Bargmann CI, Hartwieg E, Horvitz HR (1993). Odorant-selective genes and neurons mediate olfaction in C elegans.. Cell.

[27] Kaplan F, Badri DV, Zachariah C, Ajredini R, Sandoval FJ (2009). Bacterial Attraction and Quorum Sensing Inhibition in Caenorhabditis elegans Exudates.. Journal of Chemical Ecology.

[28] Stephens GJ, Kerner BJ, Bialek W, Rhu WS (2008). Dimensionality and Dynamics in the Behavior of C. elegans.. PLoS Comp Biol.

[29] Kim D, Park S, Mahadevan L, Shin JH (2011). The shallow turn of a worm.. J Exp Biol.

[30] Shenoy VB, Tambe DT, Prasad A, Theriot JA (2007). A kinematic description of the trajectories of Listeria monocytogenes propelled by actin comet tails.. Proc Natl Acad Sci USA.

[31] Zhao B, Khare P, Feldman L, Dent JA (2003). Reversal Frequency in Caenorhabditis elegans represents an integrated response to the state of the animal and its environment.. The Journal of Neuroscience.

[32] Stirman JN, Crane MM, Husson SJ, Wabnig S, Schultheis C (2011). Real-time multimodal optical control of neurons and muscles in freely behaving Caenorhabditis elegans.. Nat Methods.

[33] Hart A (2006). Behavior.. WormBook, ed The C elegans Research Community.

[34] Iino Y, Yoshida K (2009). Parallel use of two behavioral mechanisms for chemotaxis in Caenorhabditis elegans.. J Neurosci.

[35] Boyle JH, Bryden J, Cohen N (2008). An integrated neuro-mechanical model of C. elegans forward locomotion.. Neural Information Processing, Part I.

[36] Stiernagle T (2006). Maintenance of C. elegans.. WormBook, ed The C elegans Research Community.

